# Extending BioMASS to construct mathematical models from external knowledge

**DOI:** 10.1093/bioadv/vbae042

**Published:** 2024-04-04

**Authors:** Kiwamu Arakane, Hiroaki Imoto, Fabian Ormersbach, Mariko Okada

**Affiliations:** Institute for Protein Research, Osaka University, Suita, Osaka 565-0871, Japan; Institute for Protein Research, Osaka University, Suita, Osaka 565-0871, Japan; BioQuant, Heidelberg University, Heidelberg 69120, Germany; Institute for Protein Research, Osaka University, Suita, Osaka 565-0871, Japan; Premium Research Institute for Human Metaverse Medicine (WPI-PRIMe), Osaka 565-0871, Japan

## Abstract

**Motivation:**

Mechanistic modeling based on ordinary differential equations has led to numerous findings in systems biology by integrating prior knowledge and experimental data. However, the manual curation of knowledge necessary when constructing models poses a bottleneck. As the speed of knowledge accumulation continues to grow, there is a demand for a scalable means of constructing executable models.

**Results:**

We previously introduced BioMASS—an open-source, Python-based framework–to construct, simulate, and analyze mechanistic models of signaling networks. With one of its features, Text2Model, BioMASS allows users to define models in a natural language-like format, thereby facilitating the construction of large-scale models. We demonstrate that Text2Model can serve as a tool for integrating external knowledge for mathematical modeling by generating Text2Model files from a pathway database or through the use of a large language model, and simulating its dynamics through BioMASS. Our findings reveal the tool's capabilities to encourage exploration from prior knowledge and pave the way for a fully data-driven approach to constructing mathematical models.

**Availability and implementation:**

The code and documentation for BioMASS are available at https://github.com/biomass-dev/biomass and https://biomass-core.readthedocs.io, respectively. The code used in this article are available at https://github.com/okadalabipr/text2model-from-knowledge.

## 1 Introduction

Mechanistic modeling based on ordinary differential equations (ODEs) aims to elucidate the mechanisms underlying the dynamic behaviors of biological networks by integrating prior knowledge and experimental data. ODE models have been used in the field of systems biology to predict drug targets in signaling networks (Schoeberl *et al.* 2017) and stratify patients with human diseases (Imoto *et al.* 2022).

Currently, a significant amount of knowledge on the molecular mechanisms of biological phenomena has accumulated in scientific literature and publicly available databases, and such knowledge sources are expected to continue to grow. Furthermore, as the amount of high-quality omics data rises, large-scale mathematical models that can provide mechanistic insights into such data are becoming increasingly essential (Fröhlich *et al.* 2018). However, large-scale modeling can pose challenges, as the model structure can get complex, and there lies the need to manually curate existing knowledge from an extensive amount of literature, and the cost of such labor and time is enormous.

**Figure 1.**
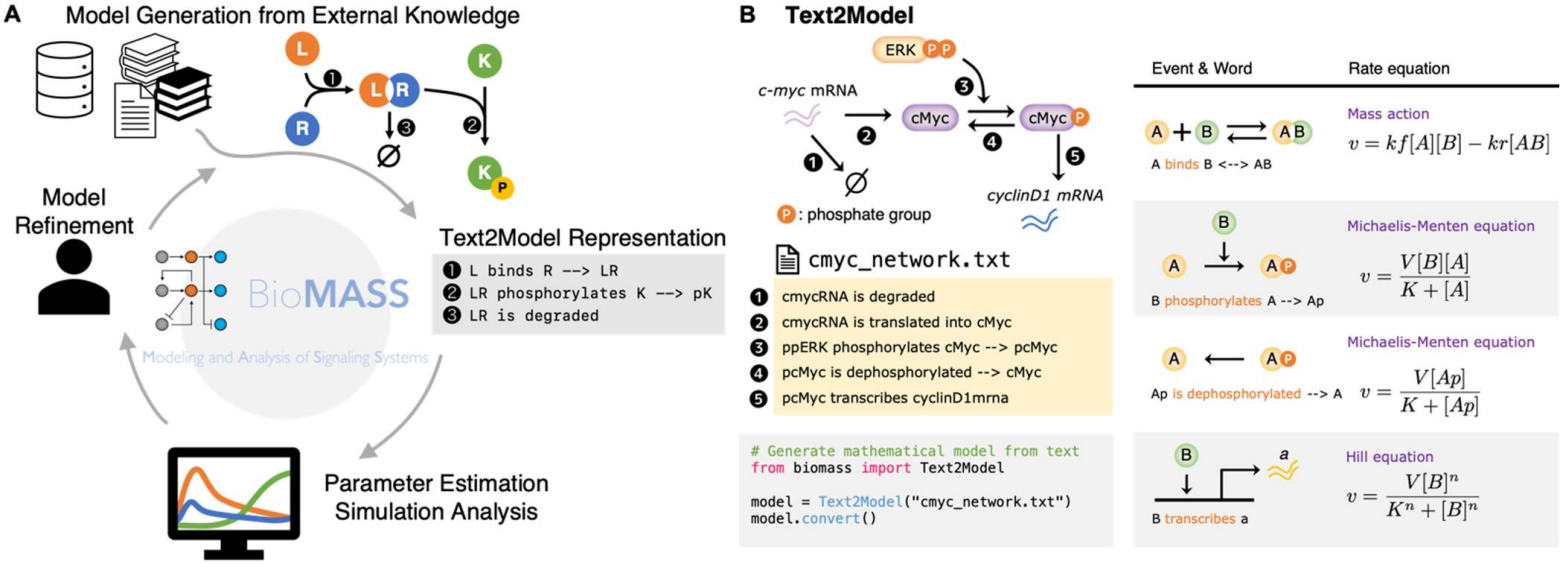
Overview of BioMASS and Text2Model. (A) Graphical abstract of BioMASS. BioMASS is an open-source, Python-based package with which users can construct and perform simulation analyses of ODE models of signaling networks. The built-in feature of BioMASS ranges from its intuitive interface to let users conduct parameter estimation and sensitivity analysis with several lines of code, to automatic detection of closed loops within the model to constrain its parameters. (B) Basic overview of Text2Model. Text2Model allows users to define a series of biochemical reactions using a format similar to natural language, thereby facilitating the construction and management of structures of large-scale models. The conversion from the Text2Model format text to a mathematical model is handled by BioMASS, which allows users to construct, simulate, and analyze mathematical models without writing a single equation. Text2Model has a predefined set of reaction types with an appropriate rate equation associated with each type; however, BioMASS allows the user to configure this list to modify associated rate equations or add new patterns.

To address this issue, we previously introduced BioMASS (Imoto *et al.* 2020), an open-source mathematical modeling platform written in Python that facilitates the construction of mathematical models, estimation of model parameters from experimental data, and sensitivity analysis through a user-friendly interface, while simultaneously providing flexibility and scalability (Fig. 1A, Supplemental Fig. S1). One of the most significant features of BioMASS is its ability to convert structured, textual descriptions of biochemical reactions (such as association, phosphorylation, and degradation) into a mechanistic model consisting of mass action equations, Michaelis–Menten equations, or Hill equations which are often used to describe signaling networks and transcriptional regulations (Imoto *et al.* 2022, Fig. 1B). This specific feature of BioMASS, named Text2Model, is intended to serve as a textual interface between biological knowledge and mathematical modeling, through which users can construct their model by defining the reactions in a format similar to that of natural language. The Text2Model formatted model is composed of human-readable lines, each corresponding to a single reaction in the model, which greatly facilitates the management of the model structure. With this feature, BioMASS enables the construction, simulation, and subsequent numerical analyses of executable models without requiring extensive knowledge of mathematics or programming, all within the same software.

The human-friendly format of Text2Model aids in the comprehension and modification of the model structure and thus makes the process of constructing, analyzing, and refining a large model more manageable, as shown in a previous study on a cancer signaling network (with 319 rate equations, 228 species, and 648 parameters) (Imoto *et al.* 2022). Other features of BioMASS include the automatic detection of closed loops within the model and constraining its kinetic parameters based on the thermodynamic principle of detailed balance. This constraint reduces the number of free variables which aids the process while preventing erroneous results from simulations and analyses. Furthermore, the tool provides a means to visualize a model as a graph, thus providing users with a more intuitive understanding of the model. With these features, BioMASS can serve as a platform for integrating experimental data which allows the user to focus on curating and testing a given hypothesis and substantially accelerating the systems biology research cycle.

However, the methods of constructing mathematical models of biological networks in BioMASS still posed a bottleneck when integrating experimental data and knowledge from multiple sources to construct a single model. Given the increasing growth of publicly accessible data and knowledge on cellular systems or gene regulation, the amount of information required to build a single model has surpassed the threshold at which each researcher can comprehensively assimilate this information.

Therefore, in this study, we propose methods to extend the capabilities of BioMASS and Text2Model to aid the process of converting external knowledge from databases and literature into executable models. We argue that, combined with BioMASS's ability to facilitate the process of model construction and simulation, our proposed method that links public knowledge domains and models using Text2Model as an intermediate language can pave the way for a fully data-driven approach towards the mathematical modeling of biological networks.

## 2 Results

In this section, we present examples of two applications in which Text2Model was used to construct executable models from external knowledge sources, and simulations were run using BioMASS.

### 2.1 Constructing mathematical models from a pathway knowledge base

Recent studies have shown that the dynamics of biological networks play a pivotal role in determining cell fate, and thus it is becoming increasingly important to be able to quantitatively understand the dynamics of the key molecules involved in this process (Shockley *et al.* 2019). Therefore, in this section, we demonstrate that it is possible to convert the general, static diagrams of biological networks from the KEGG PATHWAY database (Kanehisa and Goto 2000) into cell-specific ODE models that can describe systems dynamics through the integration of literature information using BioMASS and Text2Model.

**Figure 2.**
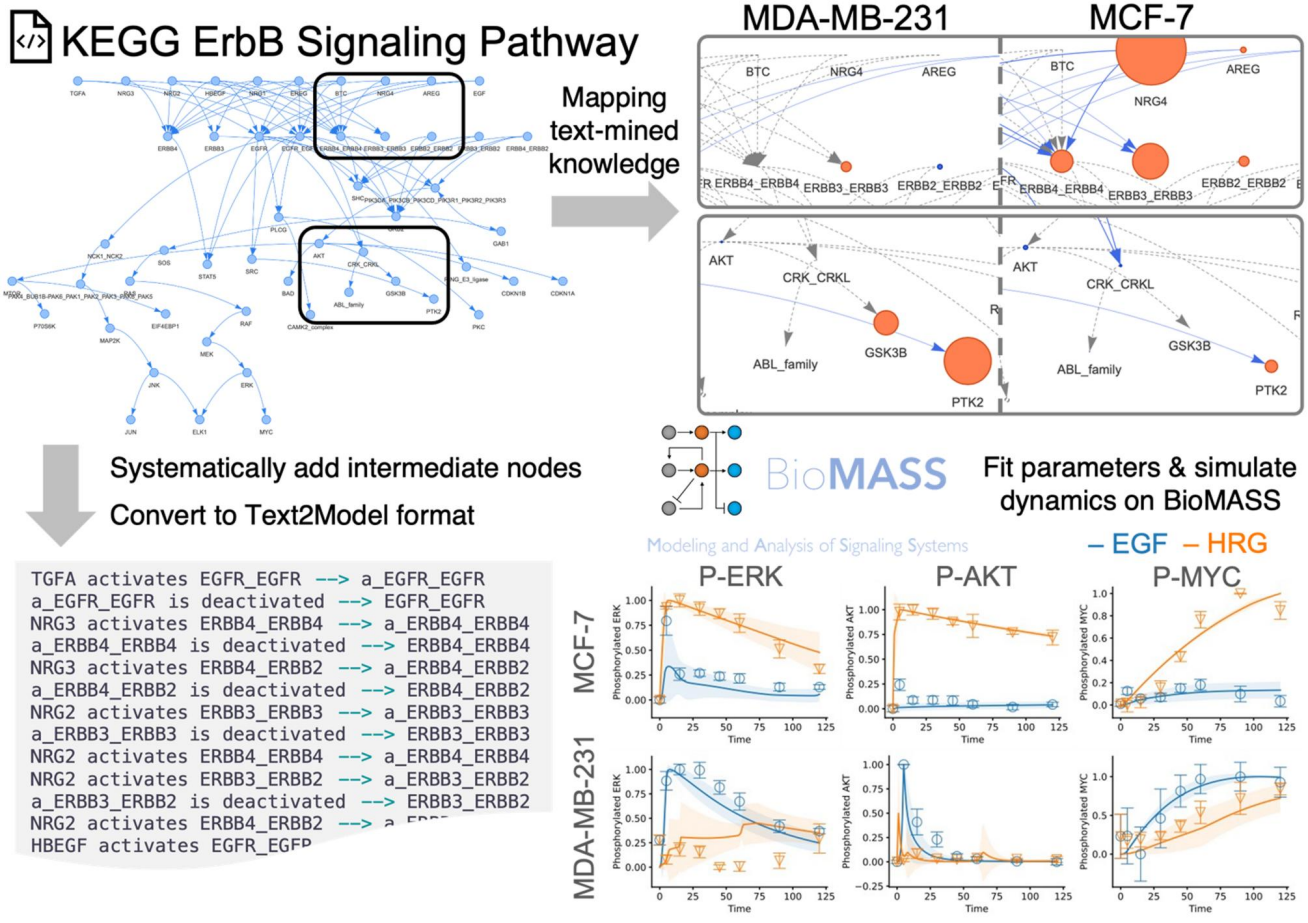
Constructing mathematical models from a pathway knowledge base. The network structure of the human ErbB signaling pathway from the KEGG PATHWAY datanase (Entry ID: hsa04012). Information regarding the nodes and edges was extracted from the KGML file (Top left). Text-mined knowledge was mapped onto the network based on specific queries (“MDA-MB-231” and “MCF-7”) to visualize and gain insight into how the network relates to a given biological term (Top right). The size of the nodes and the width of the edges represent the changes in occurrence and co-occurrence within publications, respectively, before and after applying a filter based on the given query. The extracted network is converted into the Text2Model format using the information associated with each edge (Bottom left). Simulation results of the mathematical model generated using the Text2Model file (Bottom right). The model was fitted to the experimental data for two breast cancer cell lines stimulated with the two growth factors (Imoto *et al.*, 2020). The data points in the graph denote the experimental values, whereas the lines represent the simulated values averaged across 10 parameter sets.

We developed a program to parse KEGG Markup Language (KGML) files, within which KEGG PATHWAY stores the information of each entry, and used this to obtain the structure of the human ErbB signaling pathway from the knowledge base. Using the extracted network structure, we conducted various analyses, including (a) mapping text-mined knowledge (such as the names of human diseases or cell lines) onto the nodes and edges of the network to visualize context-dependent pathways of interest and (b) semi-automatically converting the network into Text2Model and conducting parameter fitting to time-course experimental data and simulation analysis using BioMASS (Fig. 2).

In step (a), we incorporated data from PubTator Central (Wei *et al.* 2019), which provides annotated text (e.g. the names of genes, proteins, and cells) from the biomedical literature, and conducted a co-occurrence analysis of biomedical entities. By mapping the occurrence and co-occurrence of the genes within the KEGG ErbB signaling pathway to the appropriate nodes and edges, we acquired the “background” weights of each node and edge that reflect the occurrences within the entire literature. Next, by specifying a query term of interest (e.g. “MCF-7,” a breast cancer cell line), we repeated the co-occurrence analysis on a subset of the PubTator dataset which only includes publications that mention the query term. By comparing the newly calculated weights to the “background” weights, we were able to calculate the change in each gene or interaction of genes within the pathway to the given query, which we assumed to reflect the “importance” of each gene or interaction of genes within the pathway to the given query. When we compared the resulting pathway of two different query terms, “MCF-7” and “MDA-MB-231,” we observed distinct portions of the network were highlighted, indicating the possibility of distinct mechanisms within these two breast cancer cell lines. By further extending this method, it can serve as a tool to gain insight into the relationship between biological phenomena and pathway structures, which is necessary for constructing cell-specific models, in a data-driven manner.

In step (b), we used the extracted network structure to automatically generate a Text2Model file. However, several modifications were made to the network before the conversion. Because the KEGG PATHWAY database does not always explicitly contain the activated or inactivated states of the intermediate model species (e.g. phosphorylated proteins) as separate nodes within the network, the main modification was to systematically add such intermediate nodes based on the information of the reactions stored in the KGML file. After the modification, information associated with each edge was processed to generate a Text2Model representation of each reaction. During this conversion, reverse reactions for the activating reactions that were not present in the original KEGG network were automatically added to the resulting Text2Model file. Finally, to make the model more biologically relevant, we manually added reactions that represent the degradation of several model species (i.e. degradation of EGFR dimer, phosphorylated AKT, ERK, and MYC proteins) to the generated Text2Model file. The resulting Text2Model file was passed through BioMASS to convert it into a mathematical model and to conduct a simulation analysis. The model parameters were fitted to the experimental data (phosphorylation time-course of AKT, ERK, and MYC proteins) obtained from the MCF-7 and MDA-MB-231 cells stimulated with two different growth factors (EGF and HRG) that activate the ErbB receptors, which were obtained in a previous study (Imoto *et al.* 2020). The calibrated model successfully recapitulated the distinct dynamics of the two cell lines in response to various stimuli. This framework also provides a means of performing sensitivity analysis (Imoto *et al.* 2020) to identify potential drug targets for each cell line. Furthermore, another example of an application of the framework using a JAK-STAT pathway from the KEGG database is shown in the Supplementary Information (Supplementary Fig. S2).

### 2.2 Integrating BioMASS with large language models

Next, we utilized large language models (LLMs) to reconstruct mathematical models from natural language descriptions. LLMs are one of the foundations of machine learning and natural language processing (NLP) (Brown *et al.* 2020). Numerous applications of this emerging technology have been explored; therefore, we investigated the potential use cases of LLMs in mechanistic modeling. By adopting a format similar to natural language, Text2Model, as the output format of the LLM, it could be possible to reconstruct, simulate, and analyze reaction networks directly from the description of mathematical models that have been reported in publications.

To demonstrate this idea, we formulated a prompt that describes the task and expected Text2Model output in natural language, which becomes the input of the LLM. The prompt consisted of three main parts: (a) a description of the task, (b) an exemplar, and (c) the bulk of text from which the model extracted information. (a) was written completely in natural language, instructing the LLM to extract the biochemical reactions mentioned in a given passage and list them in a specified format. In (b), we included a passage that describes a sequence of reactions between fictional biomolecules, closely resembling a description of a pathway in the published literature. Following this passage, we attached a list of the reactions mentioned in the passage in the Text2Model format, which is the desired output format for this specific task. This portion of the prompt serves as an “example case” for the model to follow and is known to improve the LLM's ability to solve various tasks (few-shot prompting). In (c), a paragraph explaining an EGFR signaling pathway model from a previous study (Kholodenko *et al.*, 1999) was extracted and appended to complete the prompt.

**Figure 3.**
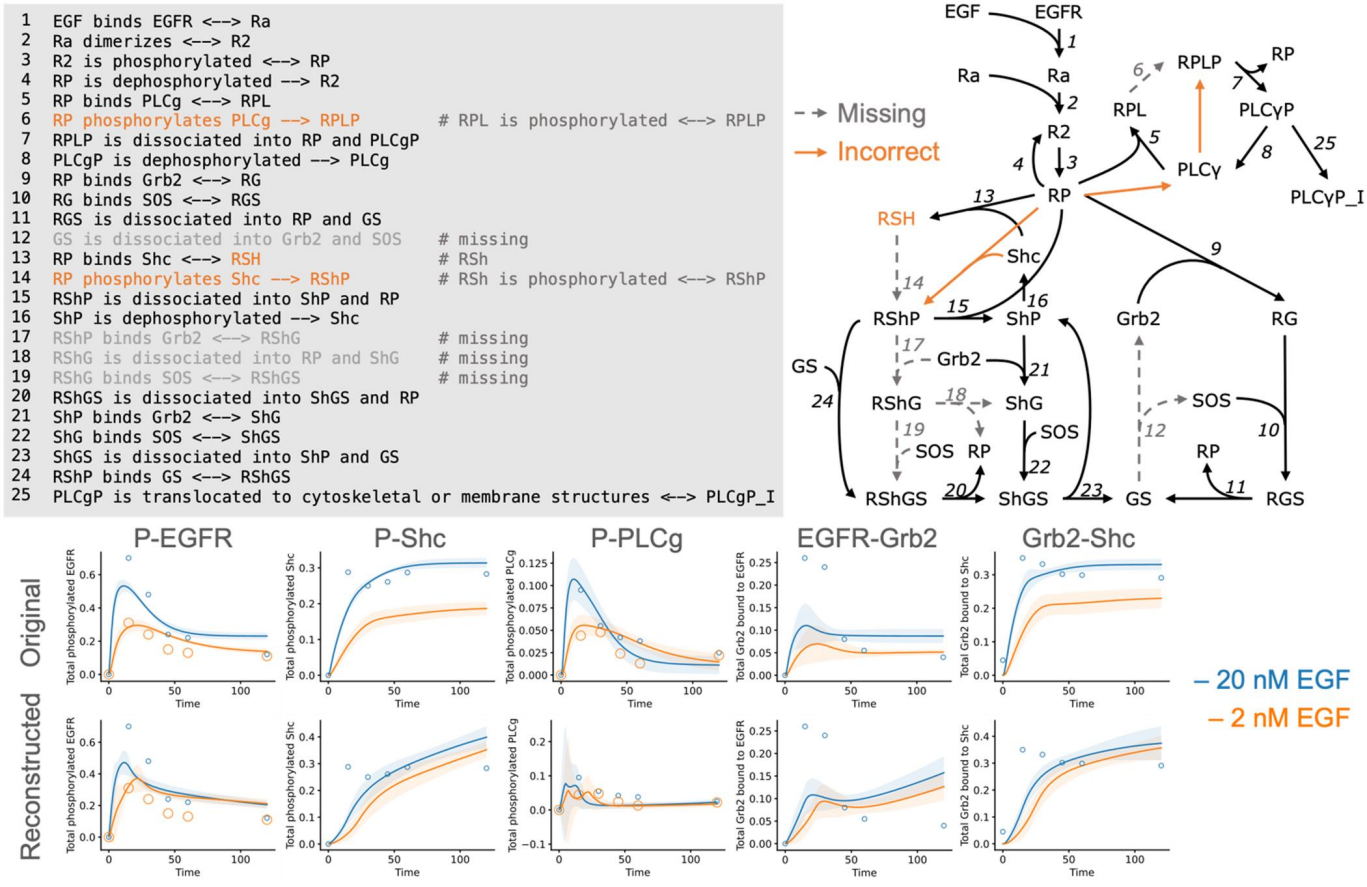
Comparison of the mathematical model reconstructed with LLMs and the original model. The Text2Model and graphical representation of the model generated by an LLM (Top). The differences compared to the human-created “ground truth” are denoted in orange (for incorrect reactions) and gray (for reactions lacking from the model) text or arrows. It is worth noting that OpenAI's LLMs are constantly being updated, and thus the result may not be reproducible in the future. Comparison of the simulation results of the “ground truth” model (original from Kholodenko *et al.* 1999) and the model reconstructed using the LLM (reconstructed) (Bottom). The parameters of both models were fitted to the experimental data provided in the original paper. The data points indicate the experimental values, whereas the lines represent the simulated values averaged across the 10 parameter sets.

Passing the prompt to an LLM (text-davinci-003, OpenAI) resulted in a valid Text2Model output (Fig. 3). However, the LLM's output lacked several reactions, including the one required to generate a thermodynamically consistent model. After the manual addition of this single crucial reaction, we converted the Text2Model output into a mathematical model using BioMASS. The parameters of the reconstructed model were fitted to the experimental data provided in the original study to assess their ability to reproduce the results of the study. Simulations with calibrated parameters indicated the ability of the reconstructed model to recapitulate the original results to a considerable extent. The generalizability of this method was tested using a JAK-STAT pathway model and the results are documented in the Supplementary Information (Supplementary Fig. S3). This result demonstrates that LLMs, in combination with BioMASS and Text2Model, have the potential to significantly automate mathematical modeling procedures.

## 3 Discussion

As a significant quantity of scientific papers and omics data have become accessible, mathematical models that can integrate such information to explain molecular mechanisms and predict important dynamics in living systems are becoming increasingly important. Large-scale executable models can provide a comprehensive means of data integration and offer interpretability; however, their construction requires significant amounts of human resources and specialized knowledge in biology, mathematics, and programming.

There have been numerous studies that attempt to overcome such bottlenecks, leading to the development of a wide range of modeling tools that provide means to access and utilize prior knowledge for mathematical modeling through human-friendly text-based or graphical interfaces. One of the most prominent examples of such tools is INDRA, with which users “assemble” pieces of knowledge collected through text mining and NLP to construct a model (Bachman *et al.* 2023). Although such tools, including BioMASS, share similar motivations, we believe they take distinct approaches to tackle the problem. Particularly, BioMASS intends to aid the process of model construction, parameter estimation, sensitivity analysis, facilitating hypothesis generation and validation without requiring mathematical or computational skills and thus has a design principle of relying on minimal external dependencies. We believe this “self-contained” approach can benefit a wide user base since it can accelerate the trial-and-error process of modeling in systems biology research. Furthermore, in this paper, we showed that incorporating external knowledge into the framework of BioMASS can further enhance its usability, demonstrating the capability of this tool to support and encourage exploration from prior knowledge.

However, the methods proposed in this paper possess some limitations, as manual correction was still necessary to refine the model generated from a knowledge base or by an LLM. The reason that such manual interventions are still necessary can be summarized as the lack of mechanistic detail in the information stored in the pathway knowledge base or included in the output of the LLM. For instance, the KEGG PATHWAY database does not explicitly include intermediate species nor the stoichiometric information regarding each reaction, and LLMs still struggle to comprehend and restrict its output based on the implicit assumptions or rules that are present when describing a chemical reaction network using natural language. Thus, it can be argued that the corrections made to the resulting Text2Model file were mainly to compensate for the lack of such mechanistic information in the outputs of the two methods.

The ambitious endeavor to fully automate the process of mathematical modeling must overcome several hurdles. Notably, extracting and integrating biological knowledge from scientific literature into the modeling scheme in a practical manner can be challenging, since the language or notation of biological reactions and molecular mechanisms are not always consistent throughout the literature and thus somehow must be normalized. Furthermore, automatically correcting the model structure and model parameters is not trivial as well, as the refinements must be based on feedback from the simulation results as well as prior knowledge to result in a biologically accurate and relevant model. With its unprecedented general task-solving capabilities, we anticipate that LLMs have the potential to overcome such problems by integrating biological knowledge and mathematical modeling. Furthermore, we argue that an “intermediate language” like Text2Model will play a vital role in leveraging this powerful NLP technology for systems biology research since it can simultaneously normalize information from unstructured data and serve as a textual interface for LLMs, which will be necessary to fully leverage the capabilities of this tool. Therefore, we believe the methods proposed in this paper represent some of the early steps towards autonomous mathematical modeling.

In addition, we acknowledge the fact that the systems biology community has put a significant amount of effort into developing and maintaining standards, such as the Systems Biology Markup Language (SBML), to ensure the reproducibility and exchangeability of mathematical models. Although BioMASS aims to be a “self-contained” framework, we resonate with the underlying motivation of this movement, and we believe implementing SBML support is of high priority and will enhance the tool's interoperability and accessibility and further contribute to the systems biology ecosystem.

Achieving a scalable method of mathematical modeling is crucial for whole-cell simulations and patient-specific modeling (i.e. avatars or digital twins) of human diseases by integrating various data from different modalities (e.g. omics data, imaging data, clinical data), and thus the methods proposed in this paper can potentially lead to personalized treatments and precision medicine.

## Supplementary Material

vbae042_Supplementary_Data

## Data Availability

The data underlying this article are available at https://github.com/okadalabipr/text2model-from-knowledge.
