# Supplementary material for: Extending BioMASS to construct mathematical models from external knowledge
Source: Bioinform Adv. 2024 Apr 4;4(1):vbae042. doi: 10.1093/bioadv/vbae042 (PMC11007111; doi:10.1093/bioadv/vbae042)
Supplement: vbae042_Supplementary_Data [file vbae042_supplementary_data.docx]

# Supplementary Information

## Overview of BioMASS

BioMASS is an open-source, Python-based package with which users can construct and perform simulation analyses of ODE models of signaling networks. The built-in feature of BioMASS ranges from its intuitive interface to let users conduct parameter estimation and sensitivity analysis with several lines of code, to automatic detection of closed loops within the model to constrain its parameters (Supplementary Fig. S1).


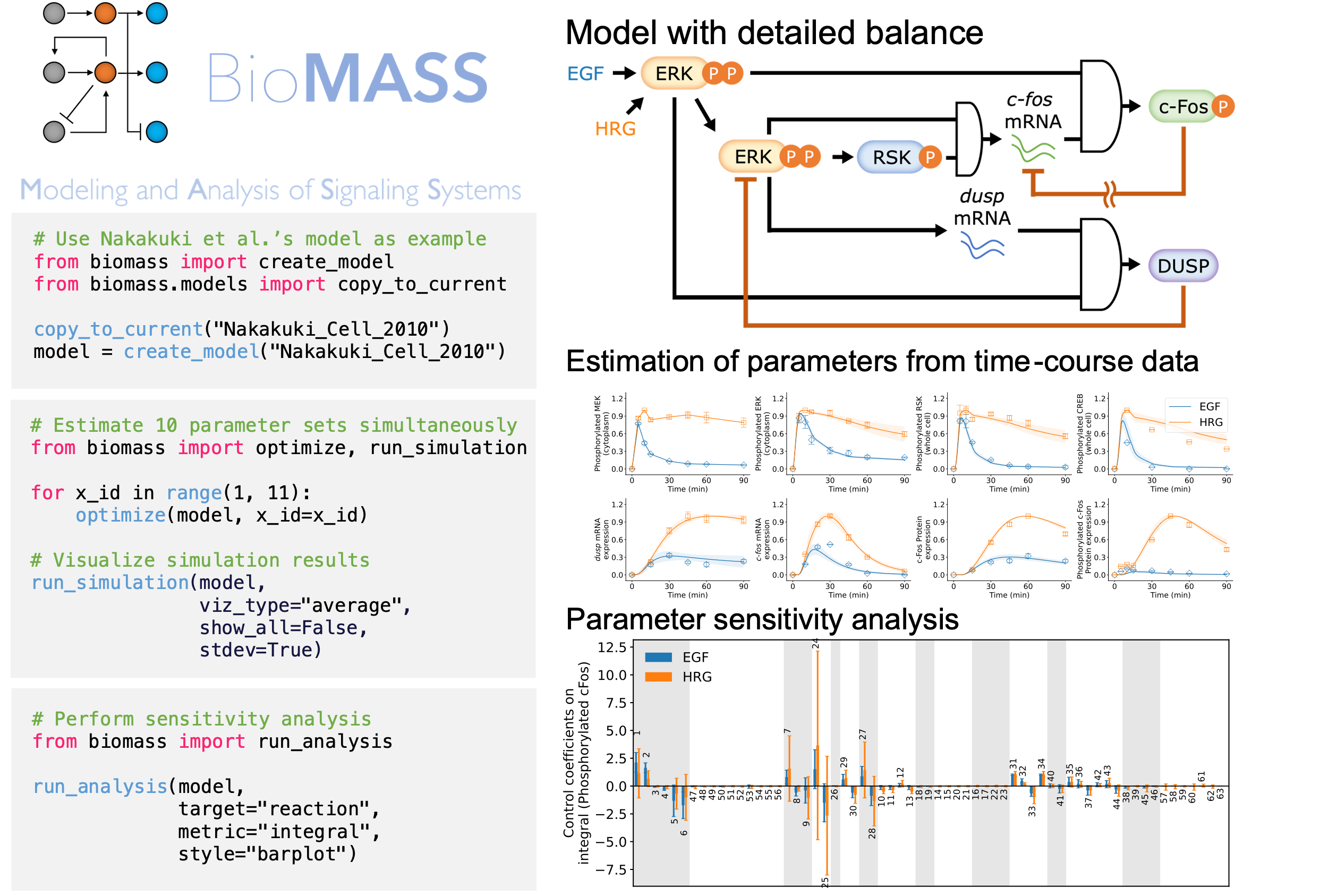


**Supplementary Fig. S1. Graphical abstract of BioMASS.** An overview of the main features of BioMASS using one of the example models included in the software **(Top right)**, which was obtained from a previous publication (Nakakuki *et al.*, 2010). The Python code to perform parameter optimization and conduct sensitivity analysis is shown. **(Left)**. When executed, the code will output the results of the parameter estimation and sensitivity analysis as images **(Bottom right)**.

## Format of Text2Model

The Text2Model file consists of mainly 3 parts: (1) the reaction, (2) observable, and (3) simulation layers. In (1), users construct their model by defining the reactions in a predefined format (see next section for the available reaction types). The reaction described in line number *i* will be converted into the *i*-th rate equation in the model. Furthermore, the initial values of the concentrations of the species and the parameter values can be explicitly set in this section as well.

In (2), users specify the species or a specific combination of species that correlate to the experimental data that is available. This can be accomplished by using the “@obs” prefix; for example, in the model used in Fig. 3, one of the observables “Total_phosphorylated_PLCg” is defined using the following line: “@obs Total_phosphorylated_PLCg: (u[RPLP] + u[PLCgP]) / (u[RPLP] + u[PLCgP] + u[PLCg] + u[RPL] + u[PLCgP_I]),” which takes the percentage of the phosphorylated form of PLCg within the total concentration of PLCg.

The time span of the simulation and the various simulation conditions (e.g., varying the initial concentration of the input or other model species) can be set in (3) using the “@sim” prefix.

For further details on the format of Text2Model, refer to the tutorial of BioMASS (<https://biomass-core.readthedocs.io/en/latest/tutorial/index.html>) as well as the documentation of Pasmopy (<https://pasmopy.readthedocs.io/en/latest/model_development.html>), in which Text2Model was originally introduced (Imoto *et al.*, 2022).

## Reaction types in Text2Model

Currently, there are 14 reaction types supported in the latest version of Text2Model, including association, phosphorylation, transcription, and degradation. A comprehensive list can be found in the online document of BioMASS (<https://biomass-core.readthedocs.io/en/latest/api/reaction_rules.html>).

Text2Model converts the given reaction types into appropriate rate equations based on a predefined table. However, there can be occasions where users wish to include a type of reaction that is not present in the list in their model. Furthermore, although the corresponding rate equations to each reaction type were decided based on careful analysis of previous examples of mechanistic models of signaling pathways, this is still only one of the many ways to model biochemical reactions, and there is no *de facto* standard of how to model each reaction type using rate equations, and this could vary between researchers or based on the biological context. In such cases, users can use the "user_defined" reaction to define the rate equation directly in the Text2Model file ([https://biomass-core.readthedocs.io/en/latest/api/reaction_rules.html#biomass.construction.reaction_rules.ReactionRules.user_defined)](https://biomass-core.readthedocs.io/en/latest/api/reaction_rules.html#biomass.construction.reaction_rules.ReactionRules.user_defined) or introduce new reaction types using the "register_word()" method (<https://biomass-core.readthedocs.io/en/latest/api/text2model.html#biomass.construction.text2model.Text2Model.register_word>).

## Experimental Data in Section 2.1

The experimental data from MCF-7 and MDA-MB-231 cells stimulated with EGF and HRG that were used in Section 2.1 were obtained from a previously published paper (Imoto *et al.*, 2020). The actual data values that were used for the parameter estimation can be found in the "observable.py" file within each BioMASS model directory ([https://github.com/okadalabipr/text2model-from-knowledge/blob/main/figure2/biomass_models/KEGG_erbb_MCF7/observable.py - L100-L130](https://github.com/okadalabipr/text2model-from-knowledge/blob/main/figure2/biomass_models/KEGG_erbb_MCF7/observable.py#L100-L130) and [https://github.com/okadalabipr/text2model-from-knowledge/blob/main/figure2/biomass_models/KEGG_erbb_MDAMB231/observable.py - L100-L130](https://github.com/okadalabipr/text2model-from-knowledge/blob/main/figure2/biomass_models/KEGG_erbb_MDAMB231/observable.py#L100-L130)).

## Manual Modifications to the Text2Model File in Section 2.1

The main modification made to the Text2Model file generated from the KEGG ErbB pathway involved defining the initial values for the parameters and the concentration of several model species, including ErbB dimers, SHC, and Ras. This information is denoted after each line of the original Text2Model file, separated with the pipe symbol (“|”).

Furthermore, the lines indicated below were appended to the file to add the degradation reactions of activated EGFR, AKT, ERK, and MYC proteins. The observable and simulation layers with lines starting with the “@obs” and “@sim” prefixes, respectively, were necessary for parameter estimation and simulation.

| # manually added lines  a_EGFR_EGFR is degraded \|kf=0.01\|  a_AKT3_AKT1_AKT2 is degraded \|kf=0.01\|  a_MAPK1_MAPK3 is degraded \|kf=0.01\|  a_MYC is degraded \|kf=0.01\|  # observable layer  @obs Phosphorylated_SHC: u[a_SHC2_SHC4_SHC3_SHC1]  @obs Phosphorylated_AKT: u[a_AKT3_AKT1_AKT2]  @obs Phosphorylated_ERK: u[a_MAPK1_MAPK3]  @obs Phosphorylated_MYC: u[a_MYC]  # simulation layer  @sim tspan: [0, 120]  @sim condition EGF: init[EGF] = 10.0; init[NRG1] = 0.0  @sim condition HRG: init[EGF] = 0.0; init[NRG1] = 10.0 |
| --- |

## Parsing KGML Files to Extract Network Structure

This section will briefly explain the steps involved in extracting the network structure from KGML files. The code for this procedure can be found in <https://github.com/okadalabipr/text2model-from-knowledge/blob/main/figure2/KEGG2Model/KGML_parser.py>.

The KGML format stores the information of the nodes in the “entry” section. Each entry in this section is parsed to extract the information of the genes or chemical compounds associated with each node. A single node can represent a group of other nodes, and such nodes are referred to as group nodes in the file.

All of the nodes are normalized and linked to an appropriate entry in an external database using Gilda (Gyori *et al.*, 2022), which was a necessary step to link the nodes in the KGML file to text-mined information acquired from the PubTator database in subsequent analyses.

The entries in the “relation” section within the KGML file contain the information of the edges. Each edge contains information regarding the reaction between the nodes, and this information was extracted and used in converting the network into the Text2Model format.

The extracted information was used to reconstruct and visualize the network structure, and external information from the co-occurrence analysis was mapped to this network. For the visualization, the Python package pyvis (<https://github.com/WestHealth/pyvis>) was used.

## Mapping Text-Mined Information onto Pathway Maps

In this section, we explain each step involved in mapping the occurrences and co-occurrences of biological entities within the literature to visualize nodes and edges relevant to specific queries shown in Section 2.1. The files that contain the code for this section can be found under <https://github.com/okadalabipr/text2model-from-knowledge/blob/main/figure2/pubtator>.

1. The entire data available from PubTator was downloaded from the official FTP server (<https://ftp.ncbi.nlm.nih.gov/pub/lu/PubTatorCentral/>). Note that the January 2022 release of the data was used in this paper, which may not be available at the time of publication and thus the results may not be fully reproducible.

For each article in the PubTator database, which can be either the abstract or full-text, the available text is split into sentences using one of the ScispaCy (Neumann *et al.*, 2019) models (en-core-sci-sm, v0.5.0). Each annotation in the sentences is processed with Gilda to associate them with an appropriate entry in an external database (normalization). This process was necessary to integrate the information acquired from the co-occurrence analysis with the KEGG pathway maps. The articles that contain 2 or more sentences with annotated entities were retained for the following analysis.

The co-occurrence analysis of the entities was conducted by counting the occasions where all pairs of entities appear within the same sentence. From this analysis, we obtain a co-occurrence matrix of the dataset, which has the size of $N \times N$ where $N$ is the number of different entities contained in the dataset. The *i,j*-th element of the matrix is the number of sentences that contain the *i*-th and *j*-th entity. For the following analysis, this co-occurrence matrix was calculated for the entire dataset (“background”) and the subset of the dataset with articles that contain the entity specified in the query (e.g., “MCF-7,” or “MDA-MB-231”).

The “background” occurrence and co-occurrence were mapped to the KEGG network using the information associated with each node. In this step, each node and edge was assigned a weight calculated from the information from the previous co-occurrence analysis. The weights were calculated by assigning the bare counts of occurrences and co-occurrences to each node and edge, respectively, and later normalized this value using the sum of the counts. The normalization was conducted separately for the node and edges. The weights calculated in this way reflect the counts relative to the entire network.

To highlight the components of the network relevant to a specific query, we first conduct the same weight-mapping analysis described in step (4), but using the query-specific co-occurrence matrix instead. We assumed the fold change between the two weights reflected the importance of each node and edge to the given query, and used this value to produce the visualization in Fig. 2.

## Converting KEGG Networks into Text2Model Files

Since the information available in the KEGG PATHWAY database is not designed for direct use in mathematical modeling, several assumptions were made to systematically accommodate for this mismatch. This section will cover the method used to convert the networks generated from the KEGG PATHWAY database into Text2Model files. The code responsible for the conversion can be found at “figure2/KEGG2Model/convert_network.py.”

1. The group nodes that are present in the original KGML file can be problematic for this specific use case since the edges that are seemingly associated with the group node according to the pathway map can either be assigned to the group node or one of its components, which can cause unexpected behaviors of the resulting model. Therefore, the conversion begins by reassigning all edges connected to any of the component nodes to the group node. This will create isolated “lonely” nodes in the network, which are subsequently removed.
2. Each edge will be processed based on the “type” value associated. For most of the event types, which are activating reactions, an “intermediate” node representing the active form of the gene will be systematically added, and all of the outgoing edges that were connected to the original node wil be reassigned to its newly created activated form. After this step, a “transition” edge will be introduced to connect the two forms to preserve the connectivity and flow of the pathway. For several exceptions, including “ubiquitination” and other inhibitory reactions, the edges are reassigned appropriately to model the inhibitory effects. One extreme exception is the “dissociation” edges, which we failed to systematically process since the use of it was inconsistent throughout the database, and will be ignored in the current method.
3. After creating the intermediate nodes and reassigning the edges, the network structure is safely converted into the Text2Model format. During the conversion, the reverse reactions of the activating reactions are systematically injected into the output. The resulting file will contain the reaction layer of a Text2Model file, and users can define initial conditions or other information necessary for simulation analyses in this file. Note that the experimental data to use in during parameter estimation must be defined in the “observerbable.py” file in the BioMASS model file, which is generated only after converting the Text2Model file with BioMASS.

## Experimental Data in Section 2.2

The experimental data used in Section 2.2 was obtained from a previous publication (Kholodenko *et al.*, 1999). Since the data values were not directly available, they were estimated from the figures in the paper. The obtained values can be found within the "observable.py" file in both BioMASS model directories ([https://github.com/okadalabipr/text2model-from-knowledge/blob/main/figure3/biomass_models/original/observable.py - L100-L121](https://github.com/okadalabipr/text2model-from-knowledge/blob/main/figure3/biomass_models/original/observable.py#L100-L121)).

## Manual Modifications to the Text2Model File in Section 2.2

The original output of the LLM only contained the information of the reactions and not the initial values of the parameters or concentrations, similar to the Text2Model file created from the KEGG PATHWAY database. Thus, this information was added as the first modification. The values used here were made identical to the “ground truth” version of the Text2Model created manually whenever possible.

The reconstructed version lacked several reactions compared to the original model, which was denoted as commented-out lines (lines starting with “#”) in the final Text2Model file. These missing lines included one of the crucial reactions needed to generate a thermodynamically consistent model (i.e., the line “GS is dissociated into Grb2 and SOS”), and therefore this specific line was manually appended to the LLM’s output.

Finally, similar to Section 4.5, the observable and simulation layers were added to relate the model to the experimental data available in the original paper. Part of these modifications are displayed below.

| # manually added lines  GS is dissociated into Grb2 and SOS \| kf=1.5e-3, kr=1e-4  #observable layer  @obs Total_phosphorylated_Shc: (u[RShP] + u[RShGS] + u[ShP] + u[ShG] + u[ShGS]) / (u[RShP] + u[RShGS] + u[ShP] + u[ShG] + u[ShGS] + u[Shc] + u[RSH])  @obs Total_Grb2_coprecipitated_with_Shc: (u[ShG] + u[RShGS] + u[ShGS]) / (u[ShG] + u[RShGS] + u[ShGS] + u[Grb2] + u[RG] + u[RGS] + u[GS])  # @obs Total_phosphorylated_Shc_bound_to_EGFR: u[RShP] + u[RShGS]  @obs Total_Grb2_bound_to_EGFR: (u[RG] + u[RGS] + u[RShGS]) / (u[ShG] + u[RShGS] + u[ShGS] + u[Grb2] + u[RG] + u[RGS] + u[GS])  # @obs Total_SOS_bound_to_EGFR: u[RGS] + u[RShGS]  # @obs ShGS_complex: u[ShGS]  @obs Total_phosphorylated_PLCg: (u[RPLP] + u[PLCgP]) / (u[RPLP] + u[PLCgP] + u[PLCg] + u[RPL] + u[PLCgP_I])  @obs Total_phosphorylated_EGFR: 2 * (u[RP] + u[RPL] + u[RPLP] + u[RG] + u[RGS] + u[RSH] + u[RShP] + u[RShGS]) / (u[EGFR] + u[Ra] + 2 * (u[R2] + u[RP] + u[RPL] + u[RPLP] + u[RG] + u[RGS] + u[RSH] + u[RShP] + u[RShGS]))  #simulation layer  @sim tspan: [0, 120]  @sim condition EGF20nM: init[EGF] = 680  @sim condition EGF2nM: init[EGF] = 68  # @sim condition Absence_PLCgP_transloc: init[EGF] = 680; p[kf25] = 0; p[kr25] = 0 |
| --- |

Note that some of the lines in the observable and simulation layers are commented out since they were not used during parameter estimation and simulations afterwards.

## Constructing an Executable Model from KEGG JAK-STAT Pathway

To test the generalizability of our method, we applied the same method used in Section 2.1 to the KEGG human JAK-STAT pathway (Entry ID: hsa04630). For the estimation of the model parameters, we used the experimental data available in a previously published paper (Raia *et al.*, 2011). Since the actual data values were not provided in the original paper, we used the same tool mentioned in Section 4.9 to estimate the experimental values from the data points. We describe each step involved below.

1. Although the initial conversion to Text2Model was successful, since the KEGG pathway map represents the general JAK-STAT pathway, the resulting file contained pathways that were irrelevant in terms of the experimental data available. Therefore, the first main modification made to the file was to remove such irrelevant species and pathways.

We noticed that, within the KEGG pathway map, the input of the model (IL13, Receptor, JAK) was represented as a group node. Due to the limitations of our method, it is currently difficult to extract the mechanistic details embedded in this format, and therefore we manually separated these species in order to increase the model’s ability to reproduce experimental data.

Furthermore, we included several crucial species and reactions, including the mRNA species of SOCS and the degradation of the receptor and SOCS, which were not explicitly included in the KEGG pathway map. The structure of the resulting model is shown in Supplementary Fig. S2.


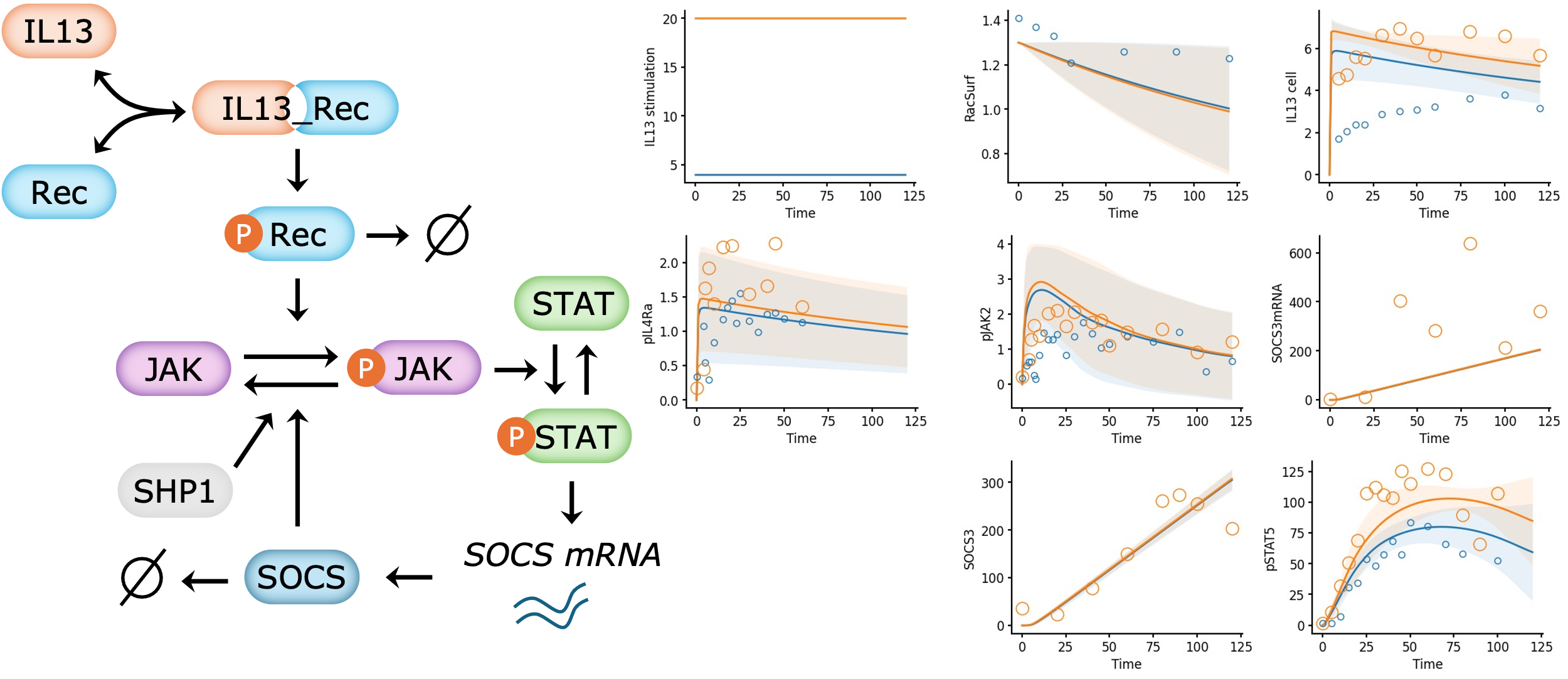
Finally, the initial values of the parameters and several species were set appropriately according to the experimental data available. The scaling values imposed in the original paper’s model were also applied to this model as well. During this step, it is worth noting that several assumptions were made regarding the mismatch between the KEGG pathway map nodes and the original model and the experimental data for specific genes were associated with the nodes that included the gene (e.g., the SOCS3 values were linked with the SOCS species in the KEGG model). See <https://github.com/okadalabipr/text2model-from-knowledge/blob/main/supplement/biomass_models/KEGG_JAK-STAT.txt> for the final Text2Model file, and <https://github.com/okadalabipr/text2model-from-knowledge/blob/main/supplement/biomass_models/KEGG_JAK-STAT/observable.py#L121-L150> for the experimental data used in this section.

**Supplementary Fig. S2. Structure and simulation result of the JAK-STAT model generated from KEGG.** The schematic representation of the structure of the converted model **(Left)**. The resulting model was relatively simple compared to the model in (Raia *et al.*, 2011). The simulation results after parameter estimation using the experimental data **(Right)**. The parameters of the model were fitted to the experimental data provided in the original paper. The data points indicate the experimental values, whereas the lines represent the simulated values averaged across 10 parameter sets.

After parameter estimation using the experimental data, the model generated from KEGG was able to reproduce the results in the original paper to some extent (Supplementary Fig. S2). However, due to the lack of several species and reactions compared to the original model (mainly the recycling of the receptor), the generated model’s reproductive powers were limited. However, as demonstrated, the textual interface provided by Text2Model makes the modification of the model structure more manageable, and the framework supports further exploration of this model.

## Reconstructing JAK-STAT Pathway with LLMs

We further tested the generalizability of the method described in Section 2.2 by applying it to reconstruct the JAK-STAT pathway model (Raia *et al.*, 2011) cited in Section 4.11. The detailed method is described step-by-step below.

1. The model description of the original JAK-STAT model by Raia et al. was extracted from the Supplementary text. This was appended to the same prompt used in Section 2.2 and was used as the input for the GPT-3 model (text-davinci-003).
2. The LLM was able to reconstruct the model in a Text2Model-like structure, however, it failed to strictly follow the Text2Model format (Supplementary Fig. S3, Top left). Similarly to the results in Section 2.2, there were several missing reactions or additional lines that were not present in the original model (denoted in gray and orange, respectively).
3. Except for the minor adjustments that were made to the model structure (i.e., adjusting the incorrect Text2Model reactions in lines 5 and 12), the output was able to be converted into an executable model. Before the conversion, the information on the initial values for the parameters and the concentration of several species, and the simulation conditions were added to the Text2Model file according to the original paper. Refer to <https://github.com/okadalabipr/text2model-from-knowledge/blob/main/supplement/biomass_models/Raia2011_reconst.txt> for the final Text2Model file, and <https://github.com/okadalabipr/text2model-from-knowledge/blob/main/supplement/biomass_models/Raia2011_reconst/observable.py#L116-L144> for the experimental data used in this analysis.
4. The model parameters were estimated from the experimental data provided by the original paper and the results were compared to the original model (Supplementary Fig. S3 Bottom).


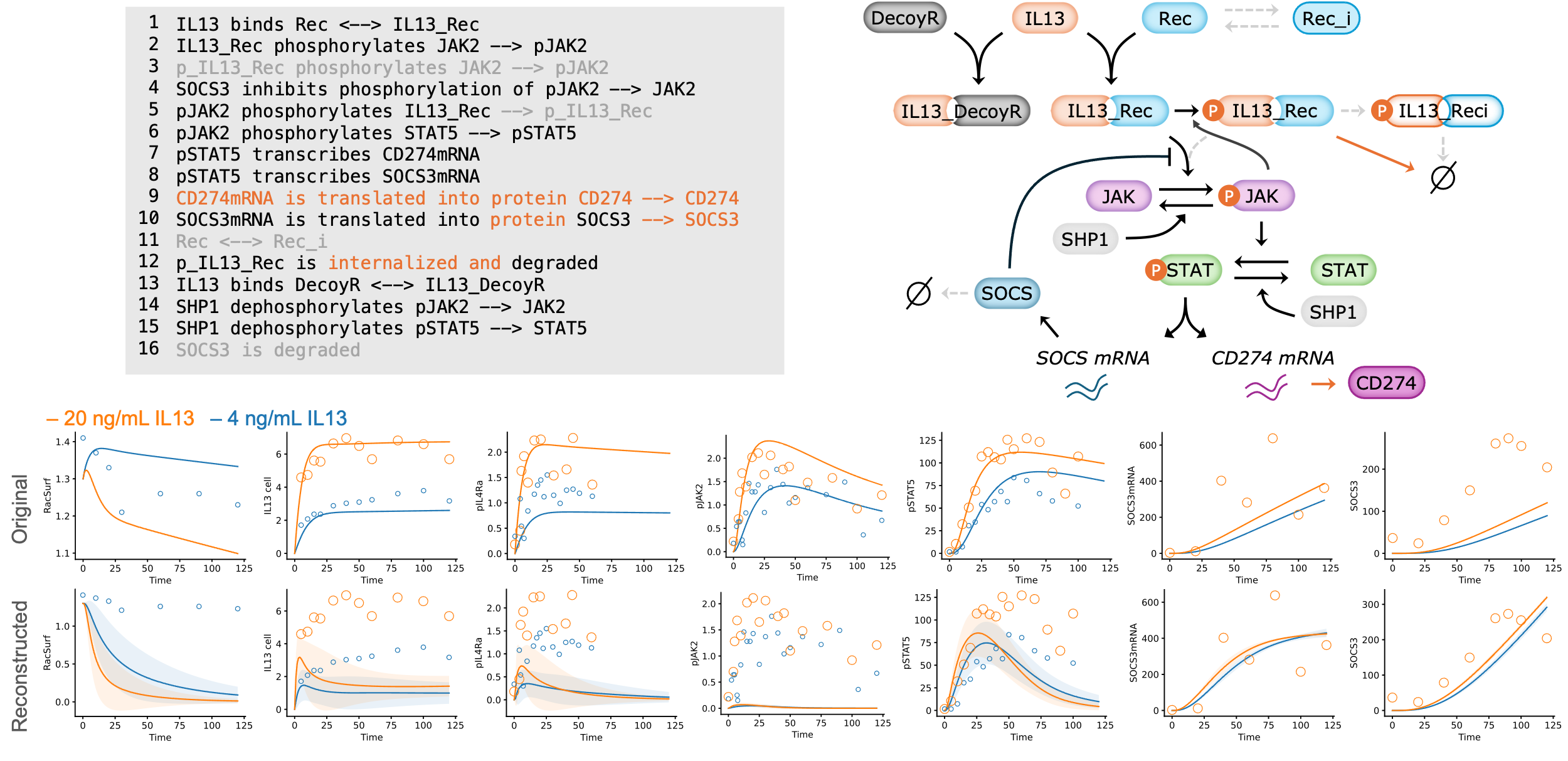
**Supplementary Fig. S3. Comparison of the JAK-STAT model reconstructed with LLMs and the original model.** The basic representation of the Text2Model format model outputted by the LLM (**Top left**). The missing and added portions compared to the manually-created “ground truth” version are denoted in gray and orange, respectively. The graphical representation of the model structure **(Top right)**. The additional and missing reactions are denoted with orange and gray, dashed arrows, respectively. Compared to the original model, species Rec_i and IL13_Reci were not present in the output, and the protein CD274 was removed since it was irrelevant to this case. Comparison of the simulation results of the "ground truth" model (original) and the model reconstructed using the LLM (reconstructed) **(Bottom)**. The original results are the simulation results using the parameter values provided by the original paper. The reconstructed results show the average trajectory of the model calibrated using the experimental data. The data points indicate the experimental values, whereas the lines represent the simulated values averaged across the 10 parameter sets.

The simulation results of the original and reconstructed model are shown in Supplementary Fig. S3. For the simulation of the original model, it should be noted that the parameter values provided in the original paper were used when applicable to the manually created Text2Model replication. Due to the differences in how the two models represent several reactions (e.g., the transcription rates of SOCS and CD274), it is not a complete replicate of the model in the original paper.

The output of the LLM was able to be converted to an executable model with minor adjustments. However, the simulated trajectories of IL13_cell, pIL4Ra, and pJAK2 deviate largely from the experimental data. Similar to the model used in Section 4.11, this is largely due to the LL failing to include the recycling of the receptor in the model (Rec <--> Rec_i). As discussed in (Raia *et al.*, 2011), this was one of the crucial reactions in the model that regulates the downstream signal intensity. Nonetheless, it is possible to add this reaction or other modifications to the model through the Text2Model interface, and the results in this section demonstrate how we can utilize LLMs to prime systems biological analyses using BioMASS and Text2Model.

Supplemental References

Gyori,B.M. *et al.* (2022) Gilda: biomedical entity text normalization with machine-learned disambiguation as a service. *Bioinformatics Advances*, **2**, 1–5.

Imoto,H. *et al.* (2020) A Computational Framework for Prediction and Analysis of Cancer Signaling Dynamics from RNA Sequencing Data—Application to the ErbB Receptor Signaling Pathway. *Cancers*, **12**, 2878.

Imoto,H. *et al.* (2022) Protocol for stratification of triple-negative breast cancer patients using in silico signaling dynamics. *STAR Protocols*, **3**, 101619.

Kholodenko,B.N. *et al.* (1999) Quantification of Short Term Signaling by the Epidermal Growth Factor Receptor. *Journal of Biological Chemistry*, **274**, 30169–30181.

Nakakuki,T. *et al.* (2010) Ligand-Specific c-Fos Expression Emerges from the Spatiotemporal Control of ErbB Network Dynamics. *Cell*, **141**, 884–896.

Neumann,M. *et al.* (2019) ScispaCy: Fast and Robust Models for Biomedical Natural Language Processing. In, Demner-Fushman,D. *et al.* (eds), *Proceedings of the 18th BioNLP Workshop and Shared Task*. Association for Computational Linguistics, Florence, Italy, pp. 319–327.

Raia,V. *et al.* (2011) Dynamic Mathematical Modeling of IL13-Induced Signaling in Hodgkin and Primary Mediastinal B-Cell Lymphoma Allows Prediction of Therapeutic Targets. *Cancer Research*, **71**, 693–704.
